# Sex differences in the association between smoking and central sensitization: A cross-sectional study

**DOI:** 10.18332/tid/175751

**Published:** 2023-12-26

**Authors:** Takeaki Takeuchi, Kazuaki Hashimoto, Akiko Koyama, Keiko Asakura, Masahiro Hashizume

**Affiliations:** 1 Department of Psychosomatic Medicine, School of Medicine, Toho University, Tokyo, Japan; 2 Department of Environmental and Occupational Health, School of Medicine, Toho University, Tokyo, Japan

**Keywords:** central sensitization, pain, sex differences, smoking, women

## Abstract

**INTRODUCTION:**

Despite the acknowledged interconnection between smoking and pain, research on the relationship between smoking and central sensitization (CS) is scarce; this pain mechanism has attracted recent research attention. Considering potential sex differences, this cross-sectional study aimed to investigate the association between smoking and CS.

**METHODS:**

Overall, 415 adult participants from an outpatient clinic underwent evaluation. The analysis focused on determining the relationship between smoking status and CS by differentiating between sexes. Data were collected on smoking presence or absence (independent variable) and CS (dependent variable) for each sex, with age, education level, drinking history, depression, and anxiety as covariates. CS was evaluated using the Central Sensitization Inventory. Following a descriptive analysis of the study population’s characteristics, logistic regression analysis was employed to assess the relationships.

**RESULTS:**

The average participant age was 42.3 years, with 59% being women. Among women, a significant association was found between smoking status and higher CS severity (AOR=3.21; 95% CI 1.29–7.99, p<0.01), after accounting for confounding variables. Conversely, no significant association was observed for men (AOR=1.50; 95% CI 0.63–3.60, p=0.36). Interaction by sex on the relationship between smoking and CS was not statistically significant (p=0.23).

**CONCLUSIONS:**

This study suggests a potential association between smoking and CS in women, whereas no conclusive relationship was observed among men. These findings indicate the necessity of considering CS when examining the relationship between smoking and pain.

## INTRODUCTION

Tobacco smoking is a major cause of various diseases and symptoms, including pain. Pain is a complex concept influenced by biological factors such as hormones and the menstrual cycle, as well as psychosocial factors such as gender roles, culture, and the perception of pain.

Previous research reports that people who smoke demonstrate increased vulnerability to pain compared to those who do not smoke^1^. Several reports present the smoking rate among pain patients to exceed that of people who do not smoke^2^. Furthermore, individuals experiencing pain, such as people who smoke, tend to smoke more frequently than their pain-free counterparts^3^. The pain leads to a craving for smoking, which has a temporary analgesic effect^4^. Smoking and pain create a positive feedback loop, reinforcing smoking behavior^4^. Furthermore, chronic smoking reduces sensitivity to pain, contributing to a vicious cycle^5^.

Sex differences are evident in pain perception, as women display greater susceptibility to pain. For somatic stimuli, females demonstrate reduced sensitivity yet enhanced discriminative captivity, higher pain ratings and decreased tolerance to noxious stimuli than males^6^. On a broader scale, the occurrence of painful disorders in women surpassed that in men^6^.

Given these facts, it is plausible that women who smoke are more susceptible to pain. While limited studies have explored the connection between smoking and pain by sex differences, earlier research has indicated that smoking heightens the probability of pain occurrence, and the effect is comparatively milder in men^6,7^. Additionally, there is a suggestion that women are more prone than men to employ smoking as a coping mechanism for pain^8^.

Recently, the concept of central sensitization (CS) has gained attention due to its role in pain perception. In CS, the central nervous system becomes markedly responsive to stimuli, potentially giving rise to enduring pain, fatigue, and other symptoms. It is characterized by the response of the central nervous system^9^. The characteristic diseases manifesting central sensitization often revolve around pain disorders, such as migraines; however, conditions like irritable bowel syndrome and chemical sensitivity disorders, which are not directly associated with pain, are also encompassed. Among these disorders, some exhibit gender differences in prevalence.

Despite the impact of CS on the relationship between smoking and pain, to our knowledge, no studies have investigated the relationship between smoking and central sensitization. Similarly, few studies have directly investigated this correlation while considering sex differences.

Therefore, we contend that assessing the relationship between smoking and CS while considering sex-related distinctions holds significance. Our hypothesis postulates a discernible association between smoking and CS, with the effect being more prominent in women compared to men.

## METHODS

### Study design and ethical considerations

This cross-sectional analysis, following the STROBE (Strengthening the Reporting of Observational Studies in Epidemiology) guidelines, was conducted at the outpatient service of the Psychosomatic Medicine Department at Toho University Hospital in Japan. This clinical study was carried out in accordance with the latest version of the Declaration of Helsinki and was approved by the Ethics Committee of Toho University School of Medicine (registration number A23059-A22086). Informed consent was obtained in the form of opt-out on the website.

### Participants and evaluation of smoking and CS

The participants included all first-time patients who visited Toho University Medical Center Omori Hospital between 1 May and 30 November 2022, and answered the questionnaire during their first outpatient visit. Patients were not selected based on age or sex (biological). However, we excluded patients whose smoking status and CS could be significantly influenced by conditions such as schizophrenia, delusional disorders, and other severe physical and mental disorders because the objectivity of responses in self-report questionnaires cannot be guaranteed. The patient’s smoking history was categorized into two groups: people who currently smoke (including those who have smoked at least 100 cigarettes in their lifetime) and people who are not presently engaged in smoking. CS was evaluated using the Japanese version of the Central Sensitization Inventory (CSI9)^10^. This contains nine symptoms pertinent to CS syndrome. Each symptom is rated on a 5-point scale ranging from ‘never’ to ‘always’. Elevated scores indicate a higher propensity for CS.

### Confounding variables

Factors such as age, education level, alcohol consumption history^11^, depression^12^, and anxiety^13^ were considered potential confounding variables in the relationship between smoking and CS. Participants were categorized into three groups based on their education level: junior high school, high school, and university education or higher. The history of alcohol consumption was categorized into two groups: regular drinking and non-regular drinking, which includes former drinking and non-drinking. Depression and anxiety levels were assessed using the seven-item Hospital Anxiety and Depression Scale (HADS)^14^. For both depression and anxiety, the scores ranged from 0 to 21 points, with the threshold for concern being 8 points for both categories.

### Sample size

We collected data from 438 first-time patients at Toho University Omori Medical Centre Hospital. No formal sample size calculation was performed for this study because this study was exploratory. The relationship between smoking and CS was evaluated using logistic regression analysis. Considering a maximum of five adjustment variables, a comprehensive sample analysis required a minimum of 50 individuals. Following the exclusion samples with insufficient data, we used data from 415 patients, consisting of 170 men and 245 women, for our analyses. The sample size was sufficient for our analysis. Consequently, we determined that the secondary analysis was methodologically acceptable.

### Statistical analysis

We conducted a descriptive assessment of the baseline characteristics of the study population concerning the primary outcome measures, namely smoking status and CSI9 score, as well as factors like age and potential confounders, including education level, alcohol consumption history, depression, and anxiety. Continuous variables are presented as means and standard deviation (SD), and differences by sex were analyzed using Student’s t-test. Fisher’s exact test (two-tailed) was employed for evaluating the differences in proportions of categorical variables.

Following a descriptive assessment of the study population’s characteristics, logistic regression analysis was employed to examine the relationship between smoking and CS, while considering differences related to sex. Regarding clinical assessment, CSI9 scores were transformed into dichotomous variables: scores <20 were assigned a value of 0, while scores of ≥20 were assigned a value of 1^8^. Statistical significance was set at a level of p<0.05 (two-tailed) for both unadjusted (ORs) and adjusted odds ratios (AORs). All analytical procedures were executed using STATA® version 14.

## RESULTS

We utilized data from 415 participants, with mean age 42.3 (SD=1.0) years, of which 245 were women. We present participants’ fundamental characteristics categorized by sex (Supplementary file Table 1). In both men and women, depression and anxiety exhibited a significant association with increased severity of clinical Central Sensitization Inventory (CSI) scores (all p<0.05). The analysis revealed a significant association between smoking status and heightened severity of clinical CSI scores in women (AOR=3.21; 95% CI: 1.29–7.99, p<0.01) (Table 1). However, no significant association emerged in men (AOR=1.50; 95% CI: 0.63–3.60, p=0.36), even after adjusting for confounding variables, including depression and anxiety (Supplementary file Table 2). Interaction by sex on the relationship between smoking and CS was not statistically significant (p=0.23).

**Table 1:** Association between smoking and clinical CSI of women in a cross-sectional study in Japan, 2022 (N=245)

*Characteristics*	*Clinical CSI*					
	*Model 1 (crude)*		*Model 2 (adjusted)*		*Model 3 (adjusted)*	
	*OR (95% CI)*	*p*	*AOR (95% CI)*	*p*	*AOR (95% CI)*	*p*
Smoking history*	3.45 (1.50–7.93)	<0.01	3.35 (1.45–7.71)	<0.01	3.21 (1.29–7.99)	0.01
Age			1.01 (1.00–1.02)	0.18	1.00 (0.99–1.02)	0.62
Education level					0.93 (0.59–1.48)	0.76
Drinking history					1.19 (0.60–2.36)	0.62
Depression					1.09 (1.01–1.17)	0.03
Anxiety					1.15 (1.05–1.25)	<0.01

* Smoking history: people who are not presently engaged in smoking (Ref.) vs people who currently smoke. AOR: adjusted odds ratio. Model 1: crude analysis. Model 2: adjusted by age. Model 3: adjusted by age, education level, drinking history, depression, and anxiety. Regarding clinical assessment, CSI9 scores were transformed into dichotomous variables: scores <20 were assigned a value of 0, while scores of ≥20 were assigned a value of 1.

## DISCUSSION

This study revealed a significant association between smoking and CS in women, while such a connection was not apparent in men. Despite the absence of direct investigations into the relationship between smoking and central sensitization, numerous studies have extensively explored the correlation between smoking and pain. The absence of a discernible association in men aligns with previous findings indicating that women are more prone to experiencing pain. Additionally, it has been postulated that women, more than men, employ smoking as a coping mechanism for pain^8^. In a previous study, sex disparity in smoking-related pain was attributed to opioid deficiency or changes in glial cell function influenced by estrogen and progesterone^15^.

Particularly in women, the potential association between smoking and central sensitization suggests possible impacts on life events specific to women and conditions related to pain. Elevated central sensitization due to smoking may contribute to adverse effects on pregnancy, childbirth, and the development or exacerbation of related disorders such as irritable bowel syndrome, chemical sensitivity disorders, chronic fatigue syndrome, and depression. Conversely, smoking cessation could be a crucial theme for future research, exploring its potential role in stabilizing pregnancy, and preventing and improving the above diseases.

### Strength and limitations

The primary strength of this study lies in its examination of the relationship between smoking and CS, accounting for sex differences. While numerous studies have explored the association between smoking and pain, research on the specific topic of smoking and CS, the focus of this study, is believed to be notably novel, to the best of our knowledge. The assessment of CS is conducted using the CSI, a questionnaire designed for evaluating CS. While the questionnaire includes symptoms other than pain, its validity as an instrument for assessing CS has been established^10^.

This study suggests a relationship between smoking and CS in women. Nevertheless, it has several limitations that warrant consideration. First, this study discusses the relationship between smoking and CS by sex; however, the interaction by sex on the relationship between smoking and CS was not statistically significant. Therefore, the association between smoking and central sensitization in females remains speculative. Second, owing to its cross-sectional design, this study cannot directly infer causality between smoking and an escalation in CS or vice versa; it can only establish a statistical association. To substantiate these findings, a more robust study design, such as a prospective cohort study, is imperative. Third, despite the inclusion of a statistically adequate number of participants in the study, the data were obtained from a single facility. The majority of participants sought treatment at the university in Japan, which limits the sample’s representativeness and the external validity of the results to other countries. Thus, further investigation involving alternative facilities is needed to generalize our outcomes. Fourth, this study constituted a secondary analysis in which smoking status was solely examined in binary terms. Research encompassing more intricate details about smoking status would likely facilitate assessments of dose-response relationships. Finally, the issue of residual confounding has not been completely resolved. Future studies should employ a more robust research design, such as a randomized controlled trial.

Nevertheless, to the best of our knowledge, no study has directly evaluated the relationship between smoking and CS while considering differences by sex. We believe that this aspect is pivotal in determining the research trajectory pertaining to smoking and pain.

## CONCLUSIONS

A potential association between smoking and CS was identified in women. These findings indicate the necessity of considering CS when examining the relationship between smoking and pain.

## Data Availability

The data supporting this research are available from the corresponding author on reasonable request.
