# Supplementary material for: Sex differences in the association between smoking and central sensitization: A cross-sectional study
Source: Tob Induc Dis. 2023 Dec 26;21:172. doi: 10.18332/tid/175751 (PMC10750460; doi:10.18332/tid/175751)
Supplement: Supplementary file 1 [file TID-21-172-s1.pdf]

Supplementary Materials

Supplemental 1. Baseline characteristics of the participants in a cross-sectional study in Japan, 2022.

	Total (n = 415)	Men (n = 170)	Women (n = 245)	p value
Age, mean (S.D.)	42.3 (1.0)	42.8 (18.2)	42.7 (20.8)	0.96
Smoking history, n (%)				<0.01
Current smoking	72 (17.3)	46 (27.0)	26 (10.6)	
Non-current smoking	343 (82.7)	124 (73.0)	219 (89.4)	
Educational history, n (%)				0.01
Junior high school	31 (7.5)	8 (4.7)	23 (9.4)	
High school	122 (29.4)	41 (24.1)	81 (33.1)	
University and over	262 (63.1)	121 (71.2)	141 (57.5)	
Drinking history, n (%)				<0.01
Regular drinking	135 (32.5)	70 (41.2)	65 (26.5)	
Non-regular drinking	280 (67.5)	100 (58.8)	180 (73.5)	
HADS anxiety, mean (S.D.)	10.0 (4.7)	9.9 (4.7)	10.0 (4.7)	0.97
HADS depression, mean (S.D.)	9.8 (5.4)	10.0 (5.6)	9.6 (5.3)	0.45
CSI score, mean (S.D.)	16.4 (7.9)	15.4 (7.8)	17.0 (7.8)	0.04
Clinical CSI score, n (%)*				0.06
High	116 (28.0)	39 (22.9)	77 (31.4)	
Low	299 (72.0)	131 (77.1)	168 (68.6)	

* A high CSI score is 21 or over, while a low score is below 20. Students' t test for continuous variables. Fisher's exact test for categorical variables.

Supplemental 2. Association between smoking and clinical CSI scores of men in a cross-sectional study in Japan, 2022 (n = 170)

	Clinical CSI					
	Model 1 (crude)		Model 2 (adjusted)		Model 3 (adjusted)	
	OR (95% CI)	p value	OR (95% CI)	p value	OR (95% CI)	p value
Smoking history	1.73 (0.81-3.73)	0.16	1.78 (0.82-3.84)	0.15	1.50 (0.63-3.60)	0.36
Age			0.99 (0.97-1.01)	0.37	0.98 (0.95-1.00)	0.07
Educational history					1.06 (0.48-2.31)	0.89
Drinking history					1.06 (0.46-2.46)	0.89
Depression					1.13 (1.01-1.27)	0.03
Anxiety					1.17 (1.03-1.33)	0.01

Smoking history (people who are not presently engaged in smoking (ref) vs people who currently smoke). Model 1: crude analysis. Model 2: adjusted by age. Model 3: adjusted by age, educational history, drinking history, depression, and anxiety.
Regarding clinical assessment, CSI9 scores were transformed into dichotomous variables; scores under 20 were assigned a value of 0, while scores of 21 and above were assigned a value of 1.

© 2023 Takeuchi T. et al.
